# Use of Attenuated Total Reflectance—Fourier Transform Infrared (ATR-FTIR) Spectroscopy in Combination with Multivariate Methods for the Rapid Determination of the Adulteration of Grape, Carob and Mulberry Pekmez

**DOI:** 10.3390/foods8070231

**Published:** 2019-06-28

**Authors:** Nihal Yaman, Serap Durakli Velioglu

**Affiliations:** 1 Department of Food Engineering, Faculty of Agriculture, Tekirdag Namik Kemal University, Tekirdag 59030, Turkey; 2 Malatya Directorate of Provincial Agriculture and Forestry, Ministry of Agriculture and Forestry, Malatya 44200, Turkey

**Keywords:** attenuated total reflectance-Fourier transform infrared spectroscopy (ATR-FTIR), grape, carob, mulberry, pekmez, adulteration

## Abstract

Pekmez, a traditional Turkish food generally produced by concentration of fruit juices, is subjected to fraudulent activities like many other foodstuffs. This study reports the use of Fourier transform infrared spectroscopy (FTIR) in combination with chemometric methods for the detection of fraudulent addition of glucose syrup to traditional grape, carob and mulberry pekmez. FTIR spectra of samples were taken in mid-infrared (MIR) range of 400–4000 cm^−1^ using attenuated total reflectance (ATR) sample accessory. Partial least squares-discriminant analysis (PLS-DA) and PLS chemometric methods were built for qualitative and quantitative analysis of pekmez samples, respectively. PLS-DA models were successfully used for the discrimination of pure pekmez samples and the adulterated pekmez samples with glucose syrup. Sensitivity and specificity of 100%, and model efficiency of 100% were obtained in PLS-DA models for all pekmez groups. Detection of the adulteration ratio of pekmez samples was also accomplished using ATR-FTIR spectroscopy in combination with PLS. As a result, it was shown that ATR-FTIR spectroscopy along with chemometric methods had a great potential for determination of pekmez adulteration with glucose syrup.

## 1. Introduction

Pekmez is a traditional Turkish food, generally produced by concentration of fruit juices in open or vacuum vessels with or without deacidification. Grape is the most widely used fruit and other fruits such as mulberry, fig and carob are also used as raw materials for the production of pekmez [1]. Because of its sugar (mainly glucose and fructose), mineral, organic acid and phenolic content, pekmez is an important traditional food in human nutrition [1,2,3].

The fruit used for the production of pekmez is of great importance due to the nutritional and technological aspects. Because of the high prices or difficulties in the supply of some fruits, as well as the high production inputs, traditional pekmez is subjected to fraudulent activities like many other foodstuffs [4]. Food products are mainly adulterated for economic gains using cheap adulterants which cause little or no change in the sensorial characteristics of the products. Nevertheless, these adulterants not only decrease the nutritional value of the product but also may threaten food safety [5]. One of the most common examples of possible adulterations made by some unlicensed producers is addition of glucose syrup to original pekmez samples and imitation of the original product appearance by addition of caramel if needed [1,4]. The addition of glucose syrup, high-fructose corn syrup or other sugars to pekmez is forbidden by Turkish Food legislation [6]. Hence, the detection of adulterants in pekmez is a crucial issue for consumers, food processors, sellers, and food authorities. There are some studies conducted for detecting the authenticity of pekmez based on the determination of 13C/12C isotope ratio via elemental Analysis-Isotope Ratio Mass Spectrometry (EA-IRMS) [4], sugars and elemental composition via enzymatic sugar analysis and atomic absorption spectrophotometry [1]. Site-specific natural isotope fractionation-nuclear magnetic resonance (SNIF-NMR) [7] and high pressure anion exchange chromatography-pulsed amperometric detection methods (HPAE-PAD) [8] were also used.

Nowadays, studies about the determination of adulterations in foodstuffs using spectroscopic methods have been extensively carried out [9] including pekmez-like high sugar containing products. Detection of honey adulteration with high fructose corn syrup and maltose syrup [10] as well as fructose, glucose, inverted sugar, hydrolyzed inulin syrup, and malt must [11] were successfully accomplished using Raman spectroscopy technique. Li et al. [12] also used one of the spectroscopic techniques, near-infrared spectroscopy, for the detection of honey adulterated with high-fructose corn syrup and maltose syrup. In a recent study, Naderi-Boldaji et al. [5] employed two dielectric spectroscopy methods for the detection of adulteration in grape syrup.

Fourier transform infrared spectroscopy (FTIR) is one of the spectroscopic methods employed for quality analysis, detection of adulteration and discrimination of food [13,14,15]. It is reported to be a faster and easier method compared to the traditional analytical methods used for the analysis of food products. It also does not require long sample preparation steps and use of chemicals which the traditionally used methods include [14,15,16,17]. FTIR can be used in conjunction with attenuated total reflection (ATR) technique for the determination of adulteration in foodstuffs. ATR is a sampling method used in combination with IR spectroscopy. It enables direct examination of the surface of soft samples and also aqueous, viscous or sticky samples. It is a faster sampling technique which provides a better reproducibility than the traditional IR sampling techniques [18].

Spectroscopic techniques are generally integrated with multivariate data analysis methods by several researchers for the determination of sugar profile [19,20], determination of geographical origin [21] and detection of adulteration [10,12,16,22,23] of honey. However, to the best of our knowledge, ATR-FTIR integrated with multivariate data analysis has not been employed for detecting the authenticity of pekmez. Hence, the aim of the present study was to utilize ATR-FTIR in combination with partial least squares-discriminant analysis (PLS-DA) and PLS methods for rapid authentication of traditional grape, carob and mulberry pekmez.

## 2. Materials and Methods

### 2.1. Materials

The samples constituted three groups, which belong to three pekmez types including grape, carob and mulberry pekmez. A total of 52 original pekmez samples (18 grape, 16 carob and 18 mulberry samples) produced traditionally were obtained directly from 30 different production plants located in Malatya, Turkey. Synthetically adulterated samples with the known adulteration levels were prepared in laboratory conditions as explained in the following subsection. Additionally, six different adulterated carob pekmez samples as test samples were obtained from the pekmez production plants in order to test the method using the real adulterated samples found in the market.

The pekmez samples were placed into amber glass bottles and kept refrigerated at 4 °C until analysis within one week.

### 2.2. Preparation of the Synthetically Adulterated Pekmez Samples

A total of 60 synthetically adulterated samples were prepared by the addition of commercial glucose syrup to original pekmez samples under laboratory conditions. Glucose syrup was selected as the adulterant due to its low price and availability.

Firstly, a master sample for each pekmez type was obtained by mixing an equal amount of original samples. Then, glucose syrup was heated up to 70 °C and mixed with the master sample at different ratios. The mass percentages used for the preparation of these adulterated samples were in the range from 2.5 to 50% (*w*/*w*) with an increment of 2.5%. This corresponded to a total of 20 adulterated samples for each pekmez type, and a total of 60 synthetically adulterated sample. The concentration range was wide to imitate the possible real-life adulteration practices.

The total soluble solids contents of the samples were determined using a refractometer in terms of °Brix values. The total soluble solids content of the samples was adjusted to standard solids content (50 °Brix) with distilled water before FTIR measurements. The spectra of the pure and adulterated samples were utilized to build PLS-DA and PLS models for the qualitative and quantitative determination of adulteration for each pekmez type.

### 2.3. ATR-FTIR Analyses of Pekmez Samples

The spectra of the samples were recorded using ATR-FTIR spectrometer (Nicolet iS50 Thermo Scientific, Waltham, MA, USA) equipped with a single bounce diamond crystal and a deuterated triglycine sulfate detector.

The FTIR spectra of samples were determined to be in the MIR range of 400–4000 cm^−1^ with a resolution of 4 cm^−1^. Each spectrum was collected from 50 scans in the absorbance mode. Triplicate measurements were made and the mean values were used. In order to obtain the FTIR spectra, the intensity values were plotted (*y*-axis) as a function of wave number (*x*-axis). OriginPro 7.5 (OriginLab Corp., Northampton, MA, USA) were used for drawing the spectra.

### 2.4. Chemometric Analysis

PLS-DA and PLS (Eigenvector Research, Inc., Wenatchee, Washington, DC, USA) were used for chemometric analyses of the FTIR-ATR data using optimal spectral data preprocessing techniques. PLS-DA was used for discrimination of pure and adulterated grape, carob and mulberry pekmez samples. After PLS-DA discrimination, PLS technique was utilized in order to determine the glucose syrup addition to various pekmez samples quantitatively.

In order to generate PLS-DA model for discrimination of pure and adulterated grape pekmez samples, 24 (12 pure, 12 adulterated) and 14 grape pekmez samples (six pure, eight adulterated) were used for calibration and validation, respectively. For carob pekmez, 22 samples (10 pure, 12 adulterated) were used for calibration and 14 samples (six pure, eight adulterated) were used for validation. For the discrimination of pure and adulterated mulberry pekmez, 24 (12 pure, 12 adulterated) and 14 samples (six pure, eight adulterated) were used for calibration and validation, respectively. In order to test the method for the real samples, the spectral data of six different carob pekmez samples obtained from the market were also added to the validation data set of carob pekmez samples.

In addition, sensitivity rate (STR, %), specificity rate (SPR, %) and model efficiency rate (EFR, %) were calculated to determine the performance of the classification [24].

STR = TPR/(TPR + FNR)(1)

SPR = TNR/(TNR + FPR)(2)

EFR = 100 − (FPR + FNR)(3)

TPR: True positive rate; FNR: False negative rate; TNR: True negative rate; FPR: False positive rate.

PLS models were generated in order to determine the adulteration ratio of pekmez samples. 14 grape, 14 carob, 12 mulberry pekmez samples and seven grape, seven carob, seven mulberry pekmez samples were used for calibration and validation data sets, respectively. Pekmez samples were randomly divided into two groups to obtain calibration and validation data sets.

The performance of the models was evaluated using coefficient of determination (R^2^), root mean square error of cross-validation (RMSECV), root mean square error of calibration (RMSEC) and root mean square error of prediction (RMSEP) values. For the PLS study, limit of detection value (LOD) and limit of quantification (LOQ) were calculated based on the standard deviation of the response and the slope of the calibration graph [25].

LOD = 3.3 × SD/S(4)

LOQ = 10 × SD/S(5)

SD: Standard deviation of the response; S: Slope of the calibration curve.

## 3. Results and Discussion

### 3.1. Brix Values of the Pekmez Samples

Total soluble solids of all pekmez samples were determined to be in the range of 69.7–76.5, 73.0–76.8, and 66.1–79.1 °Brix for pure grape, carob and mulberry pekmez samples, respectively. The values were determined in the range of 73.4–75.7, 73.3–74.6 and 71.0–73.0 °Brix for adulterated grape, carob and mulberry pekmez samples, respectively (data not shown). The total soluble solids values of the samples were adjusted to 50 °Brix before spectral analysis.

### 3.2. ATR-FTIR Spectra of Samples

**Figure 1:**
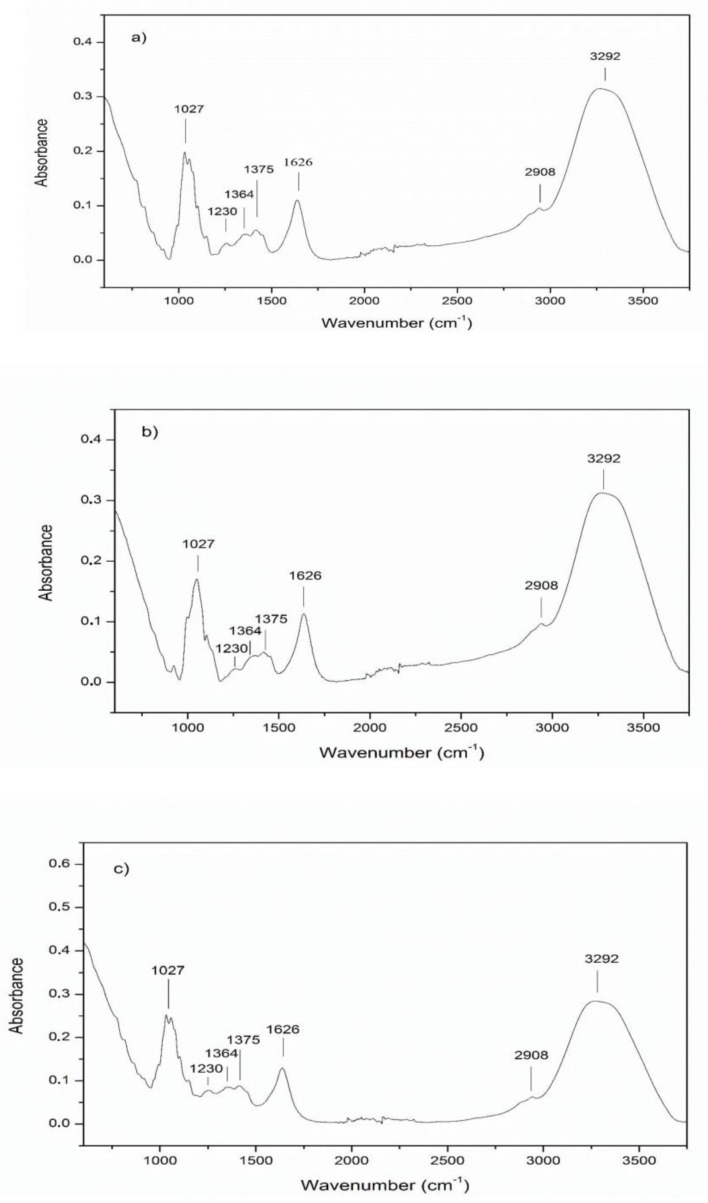
Fourier transform infrared spectroscopy (FTIR) spectra of representative grape (**a**), carob (**b**) and mulberry (**c**) pekmez samples.

The ATR-FTIR spectra of adulterated and pure grape, carob and mulberry pekmez samples were collected with FTIR spectrometer equipped with an ATR sample accessory. The FTIR spectra (400–4000 cm^−1^) of the representative grape, carob and mulberry pekmez samples are given in Figure 1a–c, respectively. As shown in Figure 1a–c, the grape, carob and mulberry pekmez samples exhibited similar absorption bands in the MIR region (400–4000 cm^−1^) as expected. However, there were some intensity differences among the pekmez types, possibly because of the differences in the composition of the fruits used in the pekmez production. The broad band located around 3292 cm^−1^ and the band at 1626 cm^−1^ were associated with O-H stretching and O-H deformation, respectively [20,22]. The occurrence of a characteristic absorption band at around 2908 cm^−1^ was associated with C-H stretching of carboxylic acid [15,20,22]. There were some absorption bands between 800 and 1500 cm^−1^. It was reported that the spectral region between n 800 and 1500 cm^−1^ would cover most of characteristic absorption bands relevant to major sugars [23]. In this region weak bands between 1200–1500 cm^−1^ were reported to be associated with the deformation of -CH_2_, and angular deformation of C-C-H and H-C-O linkages [21,22,26,27,28,29]. Intense absorption bands occurred in 950–1200 cm^−1^ were associated to C-O and C-C stretching modes of carbohydrates [22].

**Figure 2:**
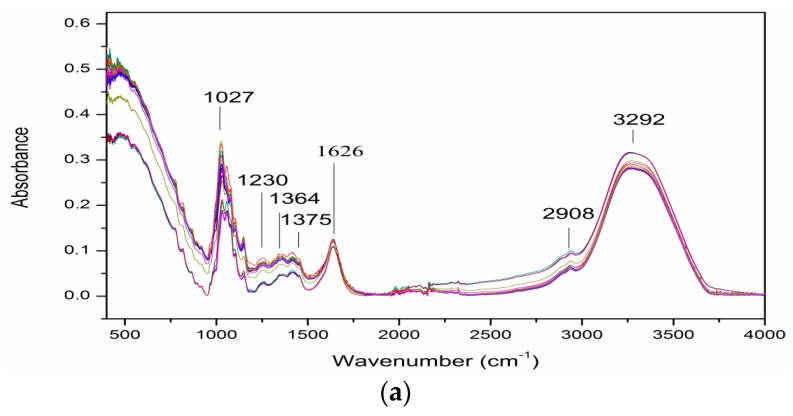

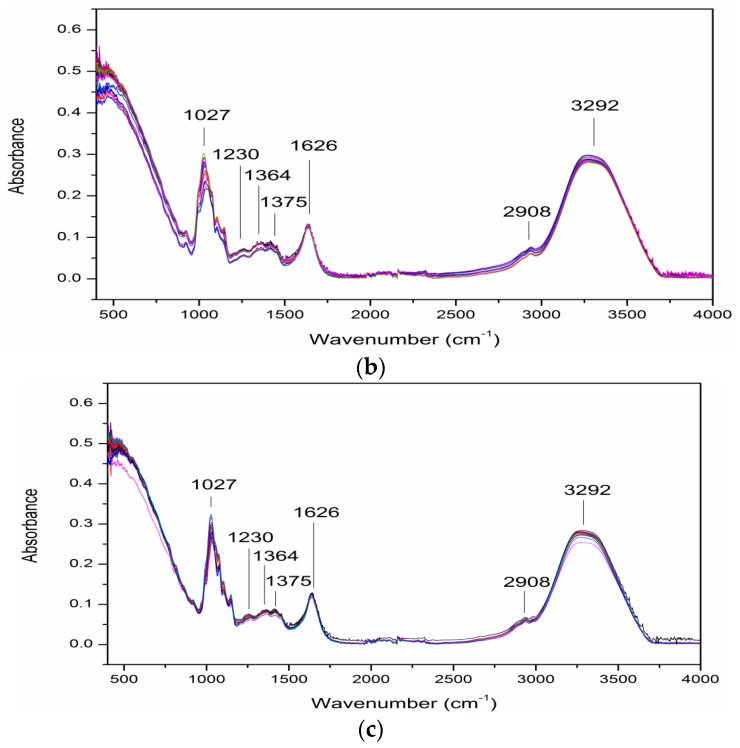
FTIR spectra of adulterated grape (**a**), carob (**b**) and mulberry (**c**) pekmez samples.

Figure 2a–c shows the spectra of grape, carob and mulberry pekmez samples adulterated with glucose syrup at different percentages (2.5–50%, *w*/*w*). The absorption of peak at 1027 cm^−1^ changed with the change in adulteration level. This peak at 1027 cm^−1^ was assigned to glucose. This finding is consistent with the findings of Se et al. [22] and Mellado-Mojica et al. [18] who reported the maximum band absorptions of glucose around 1022 cm^−1^ and 1029 cm^−1^, respectively. Glucose is present in the pekmez samples and also in the adulterant, i.e., glucose syrup in which glucose is the main sugar [1,5].

### 3.3. Discrimination of Pekmez Samples Using PLS-DA

In order to discriminate the adulterated pekmez samples, FTIR spectral data were analyzed with PLS-DA which is one of the chemometric methods. The pretreatment method having the highest performance of discrimination was determined after different pretreatments applied to the data.

**Figure 3:**
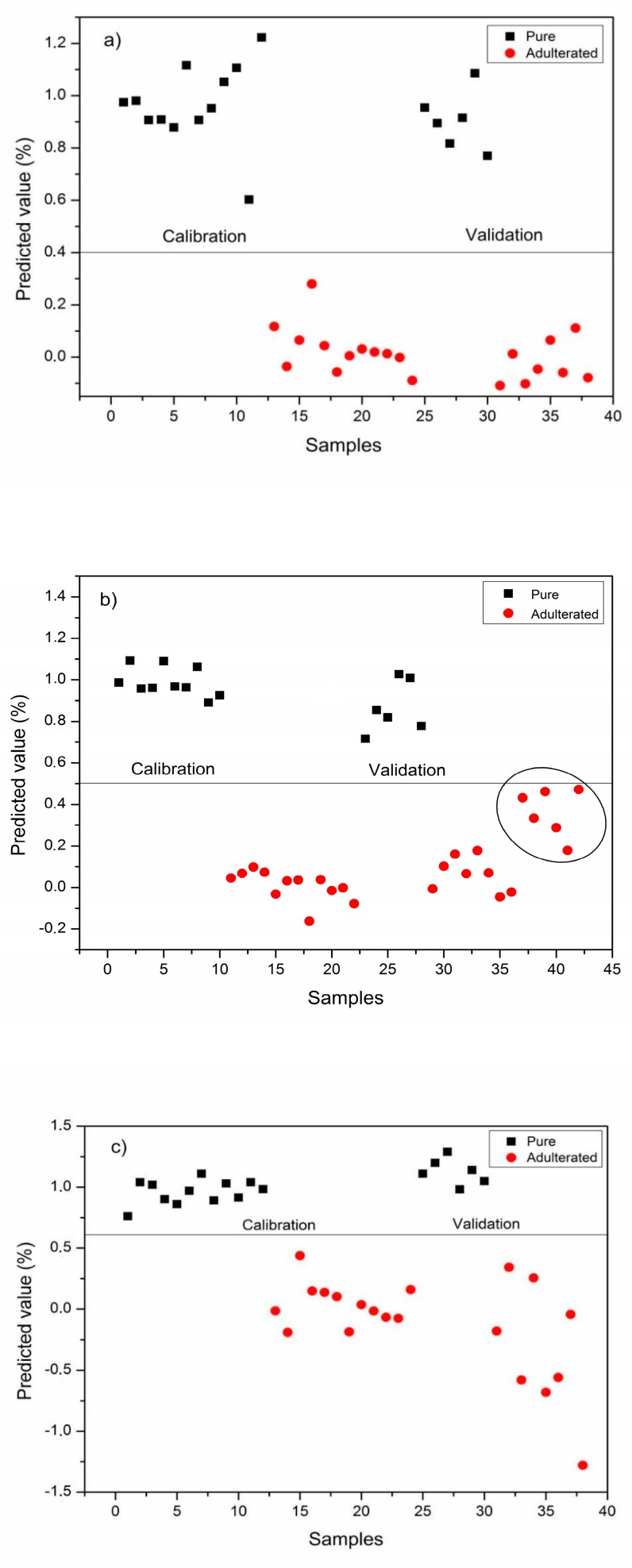
Partial least squares-discriminant analysis (PLS-DA) models for pure and adulterated pekmez samples. (**a**) Grape pekmez, (**b**) carob pekmez, (**c**) mulberry pekmez.

For PLS-DA analysis, spectral region of 600–4000 cm^−1^ were used for the discrimination of pekmez samples. As it can be seen from Figure 3, the generated PLS-DA methods successfully performed the discrimination of adulterated and pure pekmez samples for all pekmez types. The training and the test data sets were used for the calibration and validation of the models, respectively. Autoscaling was selected as a pre-process for the discrimination of pure and adulterated carob pekmez samples. First order derivatization was selected as a pre-process for mulberry pekmez samples. Normalization was selected as the pre-processing treatment for grape pekmez samples. In Figure 3b, six different samples obtained from the market were also depicted along with the validation samples. These samples obtained from the market were represented with a circle. As can be seen from Figure 3b, the adulterated carob pekmez samples obtained from the market were also discriminated from the original carob pekmez samples.

PLS-DA classification parameters for different pekmez types are seen in Table 1. According to PLS-DA results, high sensitivity (100%), specificity (100%) and model efficiency (100%) values were obtained for grape, carob and mulberry pekmez samples. These classification accuracy results were higher than the ones reported for detection of honey adulteration by NIR [10]. The results of the present study showed that the generated PLS-DA models successfully discriminated the adulterated pekmez samples (at different percentages in the range of 2.5–50%, *w*/*w*) from the original samples. The classification parameters for the three different pekmez types were similar to each other. Hence it could be reported that ATR-FTIR technique along with PLS-DA worked well in detection of adulteration in grape, carob and mulberry pekmez samples.

### 3.4. Quantification of the Adulteration of Pekmez Samples with Glucose Syrup Using PLS

The ATR-FTIR spectral data were used for determination of adulteration ratio of grape, carob and mulberry pekmez samples. The PLS calibration models were generated between the ATR-FTIR spectral data and the level of glucose syrup addition. The preprocessing method which enabled the reduction of noise in the system measurements while keeping useful variation was determined as autoscaling. Correlations between the spectral data and the adulteration level of the pekmez samples for each pekmez type were calculated. R^2^ values for the calibration and validation data sets are shown in Table 2.

**Figure 4:**
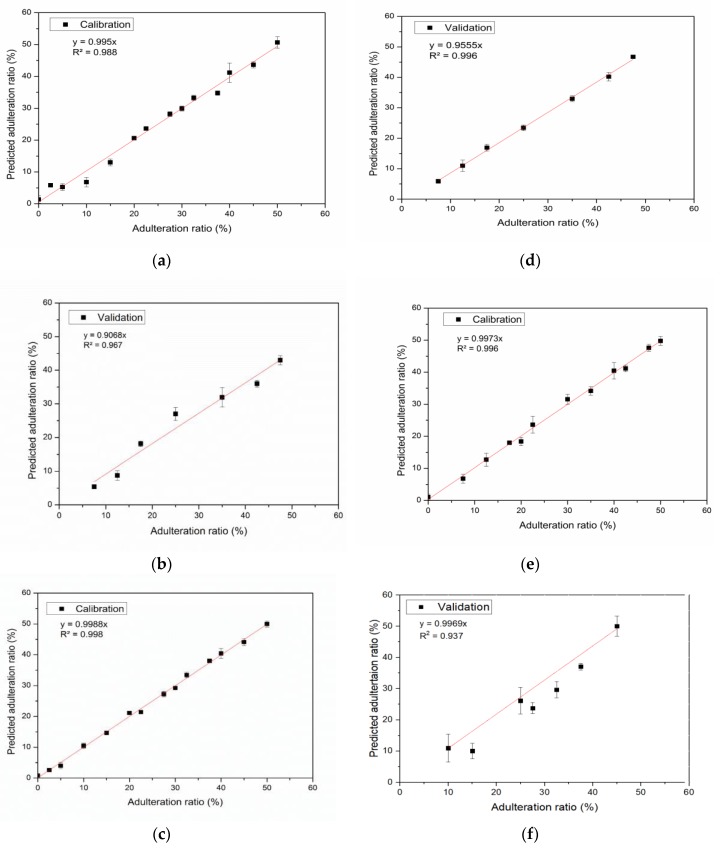
Calibration and validation data curves for grape (**a**,**b**), carob (**c**,**d**) and mulberry (**e**,**f**) pekmez samples.

R^2^ values for calibration and validation data curves except the validation data curve of mulberry pekmez were higher than 0.95 indicating a good correlation between the actual values of adulteration levels and the estimated values using PLS regression for both data sets. Figure 4 shows the calibration and validation data curves.

LOD and LOQ values are shown in Table 3. LOD values of the models were calculated as 1.33%, 2.01% and 2.99% for grape, carob and mulberry pekmez, respectively. LOQ values for the detection of the adulterations in grape, carob and mulberry pekmez samples were determined as 3.98%, 6.04% and 9.06%, respectively. These values could be regarded as satisfactory because fraudulent addition of glucose syrup to pekmez with the intent of economic gain might occur in higher levels. Se et al. [22] reported the discrimination of the honey samples adulterated with the concentrations of corn syrup above 8% (*w*/*w*) and cane sugar over 2% (*w*/*w*) using ATR-FTIR spectroscopy together with chemometrics. In the literature, detection of adulteration in foodstuffs below 10% is generally found satisfactory [22,30].

The prediction results for pekmez adulteration levels using PLS models are also seen in Table 3.

In order to assess the predictive ability of the models, RMSEC, RMSECV and RMSEP values are important as well as the coefficient of determination (R^2^) value [12]. The low RMSEC, RMSECV and RMSEP values along with high R^2^ values demonstrate satisfactory prediction results of the models. The parameters of PLS models generated can be seen in Table 3. RMSEC and RMSEP values of the models for grape, carob and mulberry pekmez were 2.017%, 0.983%, 1.618% and 3.908%, 1.856%, 10.282%, respectively. The values for grape and carob pekmez were comparable with the previous findings. Li et al. [10] reported comparable RMSEC and RMSEP values for the detection of honey adulteration with maltose syrup by using NIR spectroscopy. Basar and Ozdemir [16] also reported similar results in their study about the use of FTIR spectroscopy in combination with multivariate methods for the determination of honey adulteration with beet sugar and corn syrup. The RMSECV and RMSEP values for mulberry pekmez were higher than the ones obtained for carob and grape pekmez. These indicated that the models generated for carob and grape pekmez were more successful than the model for mulberry pekmez. The model for mulberry pekmez should be improved. Differences in the composition or in the production procedures of mulberry pekmez samples might lead to some difficulties in quantification of the adulteration in this pekmez type. Despite these differences, ATR-FTIR in combination with chemometrics enabled monitoring the adulteration in carob and grape pekmez as well as mulberry pekmez.

## 4. Conclusions

Adulteration of pure grape, carob and mulberry pekmez samples with glucose syrup was evaluated using ATR-FTIR spectroscopy. PLS-DA models were built in order to discriminate the pure and adulterated pekmez samples. Sensitivity and specificity of 100%, and model efficiency of 100%, were obtained in PLS-DA models for all pekmez types. For the quantitative analyses of pekmez adulteration, PLS models were generated. The predictive ability of the PLS models for detection of the adulteration levels in grape and carob pekmez were also good enough, however there were some difficulties in quantification of adulteration in mulberry pekmez. Nevertheless it can be concluded that ATR-FTIR spectroscopy in combination with chemometrics has a great potential to be used as a simple and fast method for the determination of adulteration of pekmez samples with glucose syrup. Further studies having larger sample sizes could be conducted in order to implement this method for routine analysis. The studies about the simultaneous determination of possible adulterants in various pekmez types using ATR-FTIR technique can also be the subject of further studies.

## Figures and Tables

**Table 1:** PLS-DA classification parameters of grape, carob and mulberry pekmez samples.

Parameters	Grape	Carob	Mulberry
RMSEC	0.128	0.068	0.141
RMSECV	0.381	0.327	0.300
STR (%)	100 ^a,b^	100 ^a,b^	100 ^a,b^
SPR (%)	100 ^a,b^	100 ^a,b^	100 ^a,b^
FPR (%)	0 ^a,b^	0 ^a,b^	0 ^a,b^
FNR (%)	0 ^a,b^	0 ^a,b^	0 ^a,b^
EFR (%)	100 ^a,b^	100 ^a,b^	100 ^a,b^

^a^ calibration; ^b^ validation; RMSECV, root mean squared error of cross-validation; RMSEC, root mean squared error of calibration; STR, Sensitivity rate; SPR, Specificity rate; FPR, False positive rate; FNR, False negative rate; EFR, Model efficiency rate; PLS-DA: Partial least squares-discriminant analysis.

**Table 2:** R^2^ values of calibration and validation curves for grape, carob and mulberry pekmez samples.

Pekmez Type	R^2^	
	Calibration	Validation
Grape	0.988	0.967
Carob	0.998	0.996
Mulberry	0.996	0.937

**Table 3:** Parameters of PLS models of grape, carob and mulberry pekmez samples.

Pekmez Type	LOD (%)	LOQ (%)	RMSEC	RMSECV	RMSEP
Grape	1.33	3.98	2.017	2.721	3.908
Carob	2.01	6.04	0.983	1.184	1.856
Mulberry	2.99	9.06	1.618	10.852	10.282

LOD: limit of detection value; LOQ: limit of quantification; RMSEP: root mean square error of prediction. PLS: Partial least squares.
